# A Rare Case of Unilateral Fetal Cataract and Coincidental Polydactyly in Congenital Toxoplasmosis

**DOI:** 10.7759/cureus.61058

**Published:** 2024-05-25

**Authors:** Harneet S Randhawa, Jasneet Randhawa, Akshay More, Akshay Jain

**Affiliations:** 1 Department of Radiology, Sassoon General Hospital, Pune, IND; 2 Department of Radiology, Government Medical College, Baramati, Baramati, IND; 3 Cardiology, Park Slope Cardiology, Brooklyn, USA; 4 Cardiology, Aulakh Hospital, Amritsar, IND; 5 Interventional Radiology, Lokmanya Tilak Municipal General Hospital and Lokmanya Tilak Municipal Medical College, Mumbai, IND; 6 Radiology, Government Medical College Kolhapur, Kolhapur, IND

**Keywords:** fgr, intracranial calcifications, polydactyly, congenital toxoplasmosis, fetal cataract

## Abstract

Congenital toxoplasmosis is caused by transplacental infection of *Toxoplasma gondii* during pregnancy. We present a case of a congenital toxoplasma with intracranial calcifications, microcephaly, growth restriction, a unilateral cataract that developed in the third trimester, and a coincidental post-axial-polydactyly. Antenatal imaging findings are important to guide further testing and confirmation of diagnosis, it is important to know all possible associations and prognoses for timely counseling, testing, and intervention. To our knowledge, no case has been published with findings of unilateral cataract in congenital toxoplasmosis and associated coincidental polydactyly. Therefore, we wish to add this case to the current scientific literature.

## Introduction

The transplacental spread of Toxoplasma tachyzoites causes congenital toxoplasmosis. The infection mostly occurs when the mother is infected during pregnancy, but it rarely occurs with pre-pregnancy infection or reactivation of infection in immunocompromised. Treating the mother with pyrimethamine and sulfadiazine may decrease transplacental transmission and reduce the severity of disease in the fetus [1]. Common imaging features described on antenatal ultrasound are ventriculomegaly/hydrocephalus, echogenic cerebral foci, periventricular abscess/echogenicity, abnormalities of the cerebellum and corpus callosum. Extra-cerebral findings include fetal growth retardation, hepatosplenomegaly, echogenic bowel, and ascites [2]. Ophthalmological findings in congenital toxoplasmosis include chorioretinitis and micro-ophthalmia. Only a few cases of fetal cataract due to toxoplasma have been described in the literature [3]. To our knowledge, no association between toxoplasma and polydactyly has been described in the literature.

## Case presentation

**Figure 1:**
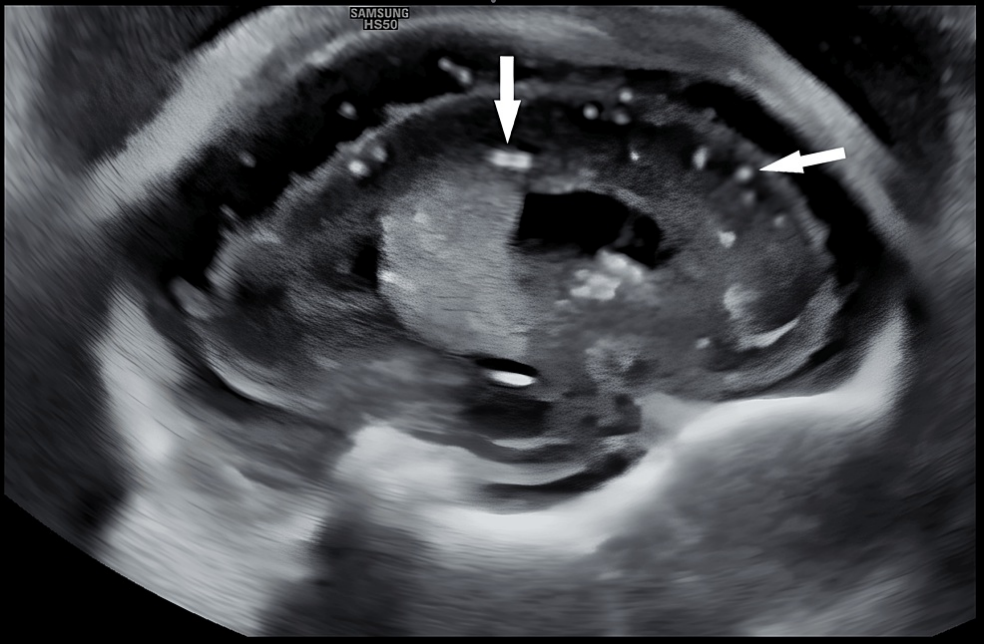
Parasagittal neuro-sonogram Echogenic foci in the cerebrum and the periventricular region as shown by white arrows.

**Figure 2:**
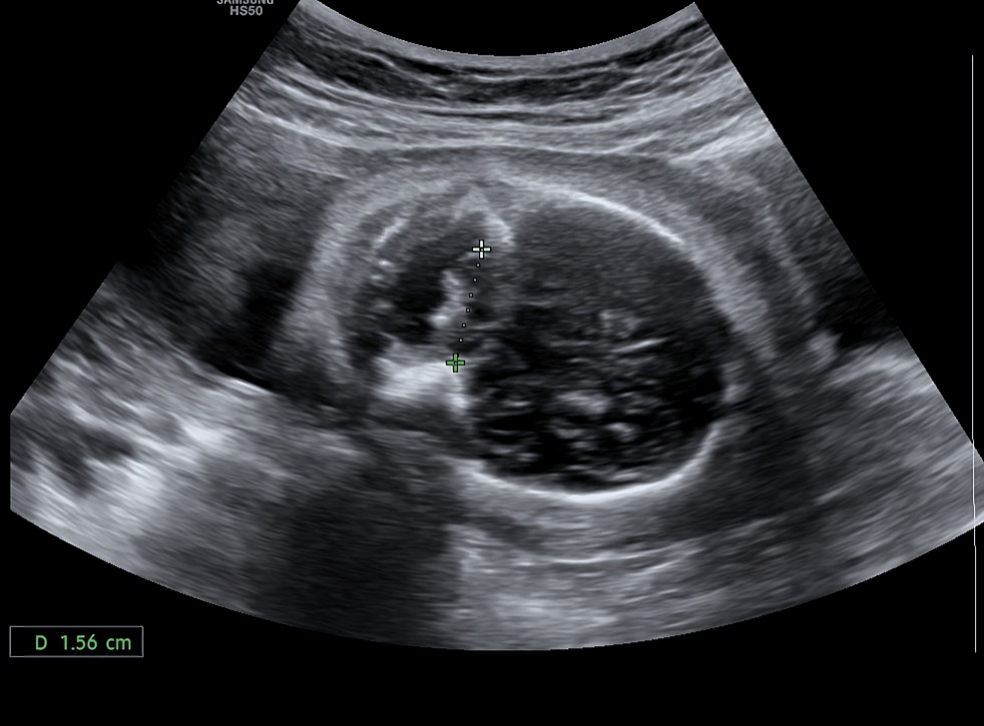
Fetal neuro-sonogram Trans-cerebellar diameter measuring 1.56 cm at 25 weeks gestation.

**Figure 3:**
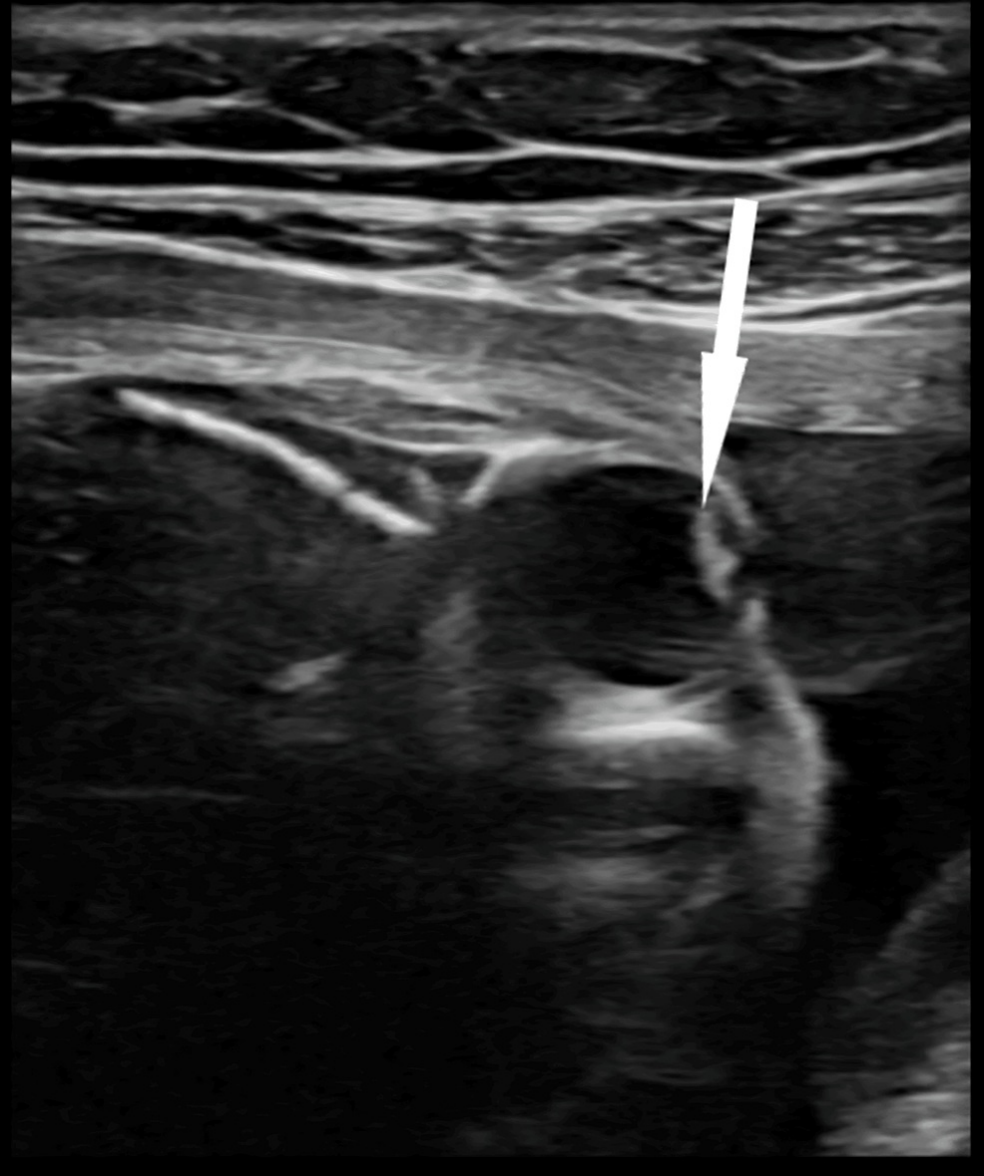
Axial section of the fetal left eye The echogenicity of the lens is suggestive of cataract, as shown by the white arrow.

**Figure 4:**
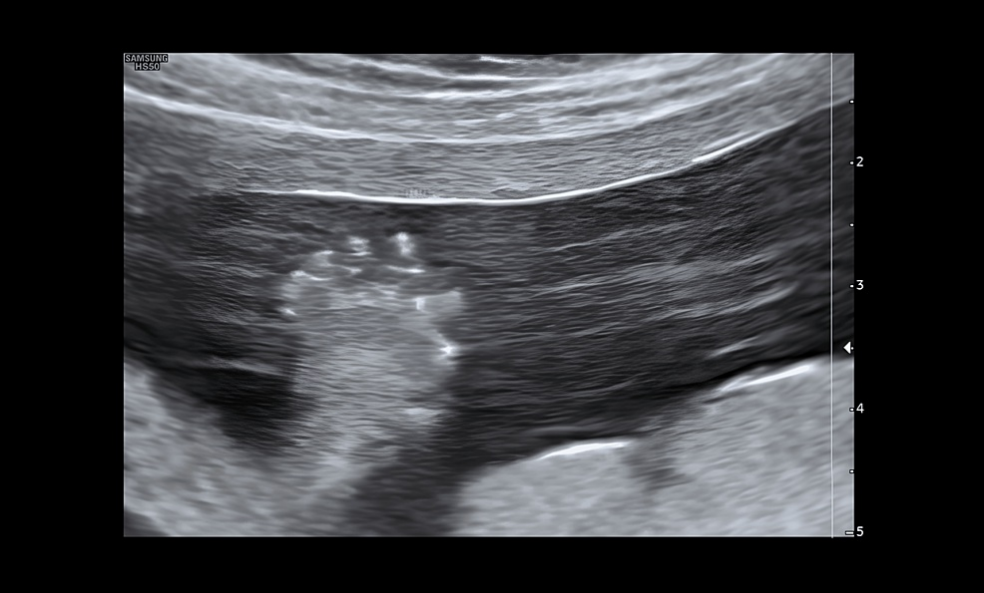
Fetal hand Left clenched fist with polydactyly

**Figure 5:**
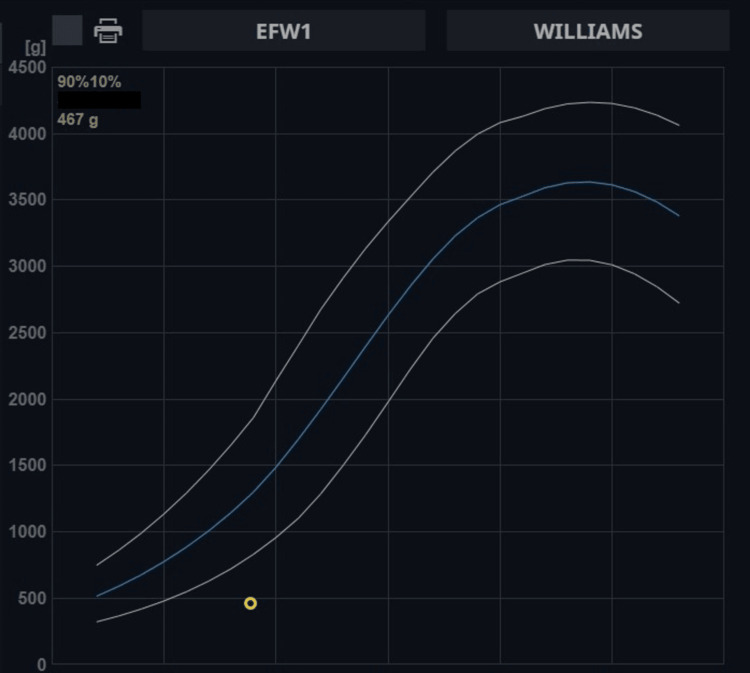
Estimated fetal weight Estimated fetal weight below the 10th percentile

A 29-year-old primigravida presented to our radiology department for an early third-trimester antenatal ultrasonography at 25 weeks gestation (gestational age assigned by last menstrual period and dating scan). The patient was afebrile and had no systemic symptoms. Her obstetric examination was suggestive of a fundal height smaller for gestational age. Her outside dating scan was normal, and a second-trimester scan at 18 weeks gestation showed growth retardation and a few echogenic cerebral foci. However, the patient had not undergone any serological studies for congenital infections. A thorough transabdominal and transvaginal scanning was done, which showed ventriculomegaly (11 mm bilaterally), echogenic cerebral and periventricular foci (Figure1), hypoplastic cerebellum (Figure2), unilateral left cataract (Figure 3), and left-hand postaxial polydactyly (Figure 4). Symmetric growth retardation was noted with head circumference, abdominal circumference, and femoral length much smaller for age and an effective fetal weight of 467 grams which was below 10th percentile for age (Figure 5).

A diagnosis of congenitally acquired infection, with the possibility of toxoplasma more likely than cytomegalovirus, was made and the patient was referred to the obstetrician for further evaluation. Serology came positive for toxoplasma, and the patient was started on pyrimethamine-sulfadiazine by the obstetrician. A detailed prognosis was discussed with the patient by a team consisting of a radiologist, an obstetrician, and a pediatrician, post which the patient decided to go for medical termination of pregnancy.

## Discussion

TORCH is the acronym used for common congenital infections, i.e. toxoplasma, syphilis, rubella, cytomegalovirus (CMV), herpes, hepatitis, and HIV. Various other infections, such as varicella and parvo B19 etc. can occur congenitally. Of these, the most common congenital infection is CMV, followed by toxoplasma [4]. Routine screening is not done for congenital infection, especially in developing and underdeveloped countries with scarce healthcare availability and unaffordability. Most fetal medicine societies only recommend TORCH serology after clinical features or antenatal ultrasonography suggests a possible TORCH infection [5].

Our patient had symmetric intrauterine growth retardation (IUGR), i.e., abdominal circumference, head circumference, biparietal diameter, and femoral length were small for gestation resulting in an estimated fetal weight less than the 10th percentile for gestational age [6]. This is mostly seen in chromosomal defects and congenital infections [7]. Our patient had calcifications in the cerebral cortex and periventricular areas. This further narrows down our differential to an infectious etiology. The typical periventricular predominant bracket calcifications (echogenicity) are seen in CMV, whereas nodular parenchymal echogenicity is more suggestive of toxoplasmosis, as seen in this case [8]. Congenital toxoplasmosis is associated with ventriculomegaly/hydrocephalous and macrocephaly, so the presence of microcephaly, as in our case, is suggestive of severe disease with poor outcome [9]. Our patient had mild ventriculomegaly suggestive of toxoplasma more than CMV. In patients with growth retardation, a small trans cerebellar diameter (TCD) is a poor prognostic feature [10], as was noted in our patient.

Cataract is mainly considered a feature of congenital rubella among infectious causes. However, cases of cataracts with toxoplasma and herpes simplex have also been reported on rare occasions. Most cases of toxoplasma cataract occur late after chorioretinitis, which is the most common ophthalmological finding in congenital infection. Only a handful of toxoplasma cases have been reported with cataracts on antenatal scans; moreover, the pathogenesis of such cases is not properly understood [11]. The presence of unilateral cataract, as in our case, is also suggestive of infective or local insult rather than a systemic cause. Therefore, in keeping with Occam’s razor principle, congenital toxoplasma was our primary diagnosis, not CMV, which was the first differential. Our patient had postaxial polydactyly which has several syndromic associations but is often sporadic [12]. However, no association has been reported with congenital toxoplasma to the best of our knowledge.

## Conclusions

Our case presents some typical features of congenital toxoplasmosis (symmetric IUGR, ventriculomegaly, and intracranial calcification), a rare finding of unilateral cataract, and a coincidental finding of polydactyly. We conclude that detailed knowledge about all possible associations and prognostic factors (small TCD, microcephaly, severity of IUGR), is important in antenatal diagnosis and management of congenital toxoplasmosis. It is also important for proper counseling of the patient about the prognosis of the child so they can make an informed decision about continuing or terminating the pregnancy. Lastly, we wish to submit the finding of polydactyly, which will add to the current scientific literature, and whether it is due to toxoplasmosis or just a sporadic finding is a matter of further study.
